# Dielectric Detection of Single Nanoparticles Using a Microwave Resonator Integrated with a Nanopore

**DOI:** 10.1021/acsomega.3c07506

**Published:** 2024-02-08

**Authors:** Arda Secme, Berk Kucukoglu, Hadi S. Pisheh, Yagmur Ceren Alatas, Uzay Tefek, Hatice Dilara Uslu, Batuhan E. Kaynak, Hashim Alhmoud, M. Selim Hanay

**Affiliations:** † Department of Mechanical Engineering, Bilkent University, Ankara 06800, Turkey; ‡ UNAM − Institute of Materials Science and Nanotechnology, Bilkent University, Ankara 06800, Turkey

## Abstract

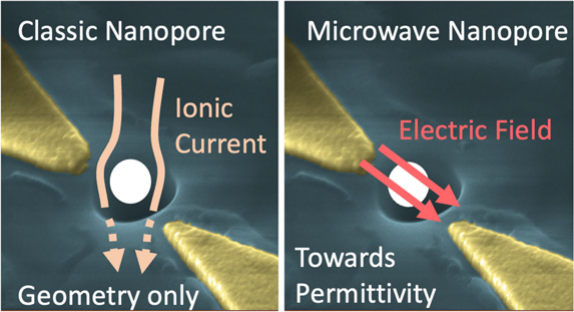

The characterization of individual nanoparticles in a liquid constitutes a critical challenge for the environmental, material, and biological sciences. To detect nanoparticles, electronic approaches are especially desirable owing to their compactness and lower costs. While electronic detection in the form of resistive-pulse sensing has enabled the acquisition of geometric properties of various analytes, impedimetric measurements to obtain dielectric signatures of nanoparticles have scarcely been reported. To explore this orthogonal sensing modality, we developed an impedimetric sensor based on a microwave resonator with a nanoscale sensing gap surrounding a nanopore built on a 220 nm silicon nitride membrane. The microwave resonator has a coplanar waveguide configuration with a resonance frequency of approximately 6.6 GHz. The approach of single nanoparticles near the sensing region and their translocation through the nanopores induced sudden changes in the impedance of the structure. The impedance changes, in turn, were picked up by the phase response of the microwave resonator. We worked with 100 and 50 nm polystyrene nanoparticles to observe single-particle events. Our current implementation was limited by the nonuniform electric field at the sensing region. This work provides a complementary sensing modality for nanoparticle characterization, where the dielectric response, rather than ionic current, determines the signal.

## Introduction

Label-free detection of nanoparticles in a liquid holds a key position for many applications in biology, materials science, and environmental engineering. Nanoscale sensing techniques based on optical resonators,^1,2^ suspended nanochannel resonators,^3^ and resistive-pulse sensing across a nanopore^4^ have all demonstrated the sizing of nanoparticles inside a liquid. Among these techniques, entirely electronic techniques, such as resistive-pulse sensing across a nanopore, offer the advantage of parallelism, low-cost, and portability. For important biological applications such as nucleotide and protein sequencing, nanopore-based techniques have already achieved commercialization^5^ and proof-of-concept demonstrations,^6−12^ respectively. Resistive-pulse sensing with nanopores^13^ has also been used to detect single nanoparticles^4,14^ and viruses.^15−17^

**Figure 1:**
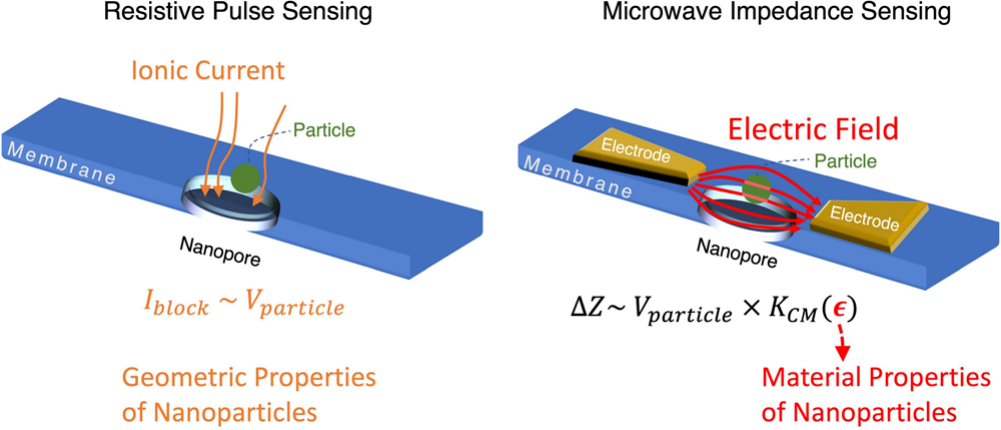
Motivation for microwave nanopore sensing. In typical resistive-pulse sensing scenarios (left panel), the current across a nanopore is measured, which gets blocked partially by a dielectric particle passing through the nanopore. Since the blockage current depends only on the size and shape of the particle, resistive-pulse sensing does not directly provide information about the internal properties. Right panel: at high frequencies, a nanoparticle can be probed through the impedance change it induces across two electrodes—which are part of a microwave resonator in this study (the microwave resonator is not shown here). This impedance change can be related to the real and imaginary parts of its dielectric permittivity, through the Clausius–Mossotti factor *K*_CM_.

While resistive-pulse sensing is an invaluable technique, it can be complemented by additional electronic measurements conducted on the same particle.^18^ In resistive-pulse sensing, the ionic current through a nanopore is partially blocked by an insulating particle as the particle passes through the nanopore. Since the magnitude of the current blockage depends on the geometric size of the nanoparticles—and not on their material properties as long as the particles are insulating—particles of different composition but similar size result in similar signals. Therefore, to obtain material-specific information in nanoparticle applications, complementary measurements are needed such as the dielectric response of the nanoparticles. This way one can combine both geometric size and capacitive size measurements to obtain the dielectric permittivity of a material and hence perform permittivity-based material classification of nanoparticles^19^ (Figure 1).

In principle, the dielectric response of nanoparticles can be detected by a capacitive sensor with a nanoscale sensing region at any given AC frequency. In practice, however, the operation of a capacitive sensor in liquid is hampered at low AC frequencies by Debye screening effects on the material interfaces, such as at the electrodes and on the surface of nanoparticles.^20^ A potential remedy is to operate the electronic sensor at microwave frequencies to overcome such screening effects.^21,22^ Indeed, a scanning probe sensor with a microwave resonator (in air) has been shown to detect nanoparticles and obtain their dielectric signals.^23^ Inside a liquid, scanning microwave microscopy has accomplished the imaging of internal bacteria structure^24^ and cells obstructed by a thin membrane.^25^ Compared with probe-based microwave techniques, chip-based techniques offer direct integration capability with microfluidic platforms. The chip-based approach of interfacing a micropore with a microwave device was recently demonstrated in ref (22) for detecting 500 nm beads, which employed different measurement and fabrication techniques compared to those reported here. In this work, we demonstrate that single nanoparticles of 50 and 100 nm diameter can be detected capacitively by on-chip microwave resonators fabricated in the proximity of a solid-state nanopore. In the following, we first describe the device paradigm and experimental system followed by the detection of 100 nm polystyrene particles with this platform and their statistics.

## Results and Discussion

### Experiments

**Figure 2:**
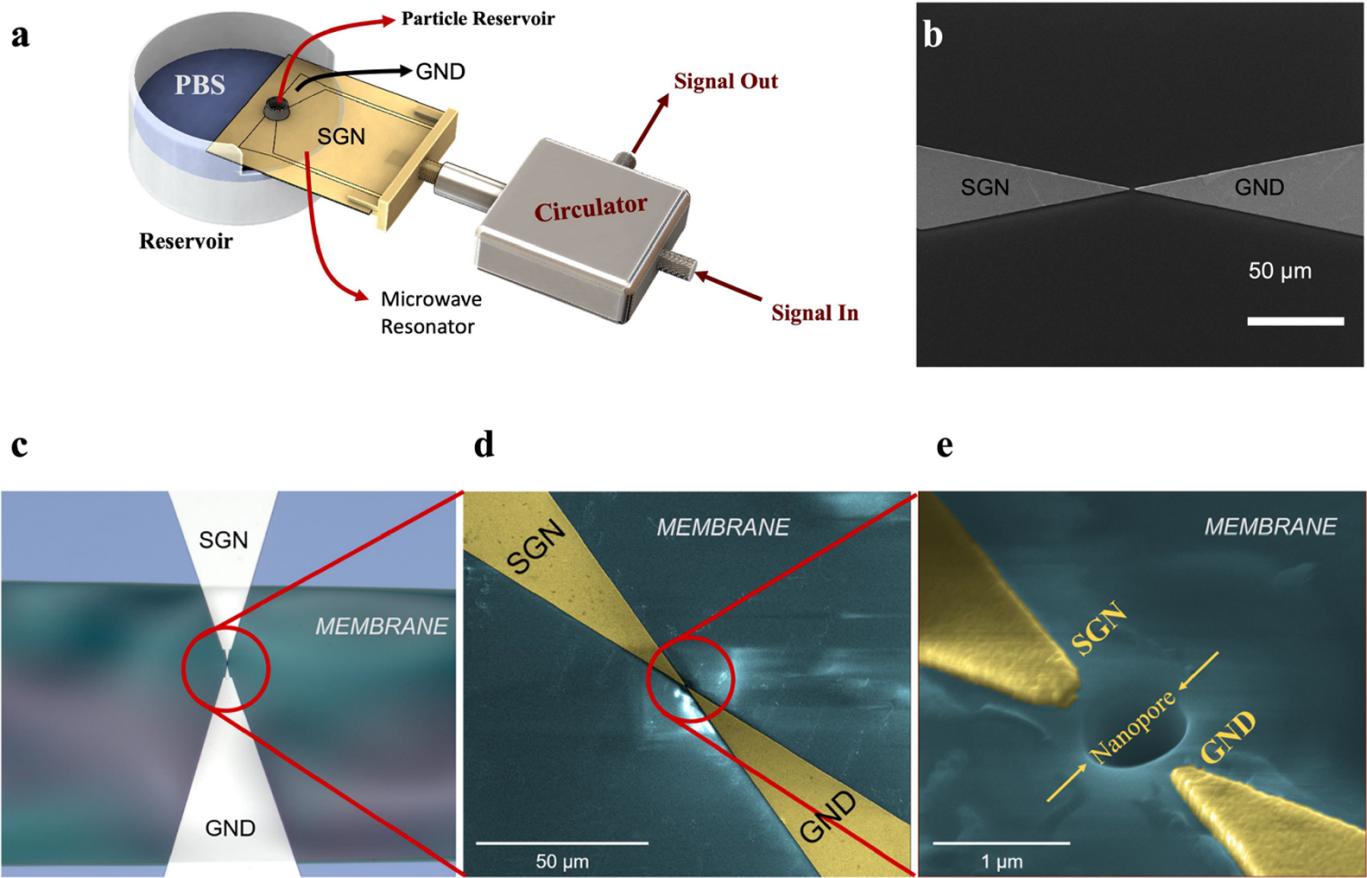
Experimental system and the device. (a) Setup consists of two reservoirs and a microwave resonator in the form of a coplanar waveguide resonator. The signal (SGN) and ground (GND) electrodes of the resonator converge on a membrane. The particle reservoir is aligned with the membrane as well. (b) SEM image of the sensing region before the membrane and nanopore were fabricated. (c) Optical microscopy image of the membrane and the sensing region. (d, e) SEM images of the sensing region and the nanopore (tilted) at successive resolutions.

The fabricated sensor is shown in Figure 2. We used a coplanar waveguide microwave resonator where the active sensing region is fabricated on a membrane, so that a nanopore can be drilled readily. The sensor was fabricated on a commercial wafer consisting of a 500 μm-thick Si substrate and two additional material layers. The first layer was 2.2 μm thick SiO_2_ on top of Si, and the second (outermost) layer was a 220 nm-thick Si_3_N_4_ layer. The wafer contained SiO_2_ and Si_3_N_4_ layers on both sides. During the fabrication (SI Section 1), we first processed the top side of the chip by performing electron beam lithography followed by metal deposition to define the narrow sensing region where two metal electrodes approach each other with a gap of approximately 600 nm. This step was followed by photolithography and metal deposition to define the larger features of the microwave sensor in the form of a coplanar waveguide. For both lithography steps, 10 nm Cr and 100 nm Au were deposited as the metallization layer.

After the process on the top surface was completed, we proceeded with membrane fabrication.^26^ A window was opened at the bottom of the wafer with anisotropic etching of Si_3_N_4_ to enable backside KOH wet etching. Si and SiO_2_ were etched by KOH until the top Si_3_N_4_ layer was suspended, which forms the thin membrane. Care was taken to align the backside window with the sensing region at the top as much as possible; this way, the narrow sensing region at the top surface sits on the Si_3_N_4_ membrane. In the last step, focused ion beam was used to drill a hole on the membrane in between the sensing electrodes. In the final configuration, electrodes were aligned on the membrane (Figure 2c,d) and a nanopore was opened between the electrodes, having typically 450 nm diameter and 220 nm thickness determined by the thickness of Si_3_N_4_.

The fabricated chip was placed in a test setup inside a Faraday cage. The test setup contained a liquid reservoir with phosphate-buffered saline (PBS). The chip was partially submerged into the liquid reservoir at a distance that covers the bottom etching window completely (Figure 2a), while keeping the microwave readout port dry for external electronic access. A small liquid chamber called the particle reservoir was attached to the top side of the chip and aligned with the sensing region so that the nanopore area is covered by the particle reservoir. Each reservoir was supplemented with a wire to induce electrokinetic flow of nanoparticles between the reservoirs and through the nanopore (the zeta potential is extrapolated to be between −50 and −60 mV, which implies electroosmotic flow). A sourcemeter (Keithley 2401) was used to measure the electronic characteristics between the two wires. One of the wires was placed inside the large reservoir, where the sensor was also partially submerged, and the other wire was placed inside the particle reservoir (Figure S2). Current–voltage measurements between the two wires were conducted using the sourcemeter to verify that the two reservoirs were connected via the liquid that contains ions. The linearity of the *I*–*V* curves was established for the voltage ranges used in the experiments (Figure S2).

The microwave resonator used in the experiments was measured by a heterodyne mixing circuitry, also known as a microwave interferometer.^27−30^ Owing to the frequency down-conversion of the signal, phase-sensitive detection using lock-in amplifiers in the 5 MHz range was employed. Further details of the measurement circuitry are described in SI Section 3. The first set of experiments was performed with polystyrene (PS) nanoparticles with a 100 nm nominal diameter (Sigma Aldrich 43302). The original samples were diluted with PBS by 60-fold. Aliquots of a 50 μL volume of the diluted solution were added to the top reservoir, located on the top surface of the chip. For the sensor used in the experiments, the electrodes were separated by 630 nm, and the nanopore had a diameter of 450 nm. The resonance frequency of the device was at 6.62 GHz: the operation frequency was chosen so that it is fast enough to avoid the electrical screening by solute ions but avoids the water relaxation process which peaks around 10 GHz. The resonance was tracked with a sampling rate of 50 kSa/s and lock-in time constant of 50 μs (which corresponds to a 3 dB bandwidth of 3.2 kHz with a first-order filter). These parameters were chosen to temporally resolve single events that occur within a duration of several milliseconds.

**Figure 3:**
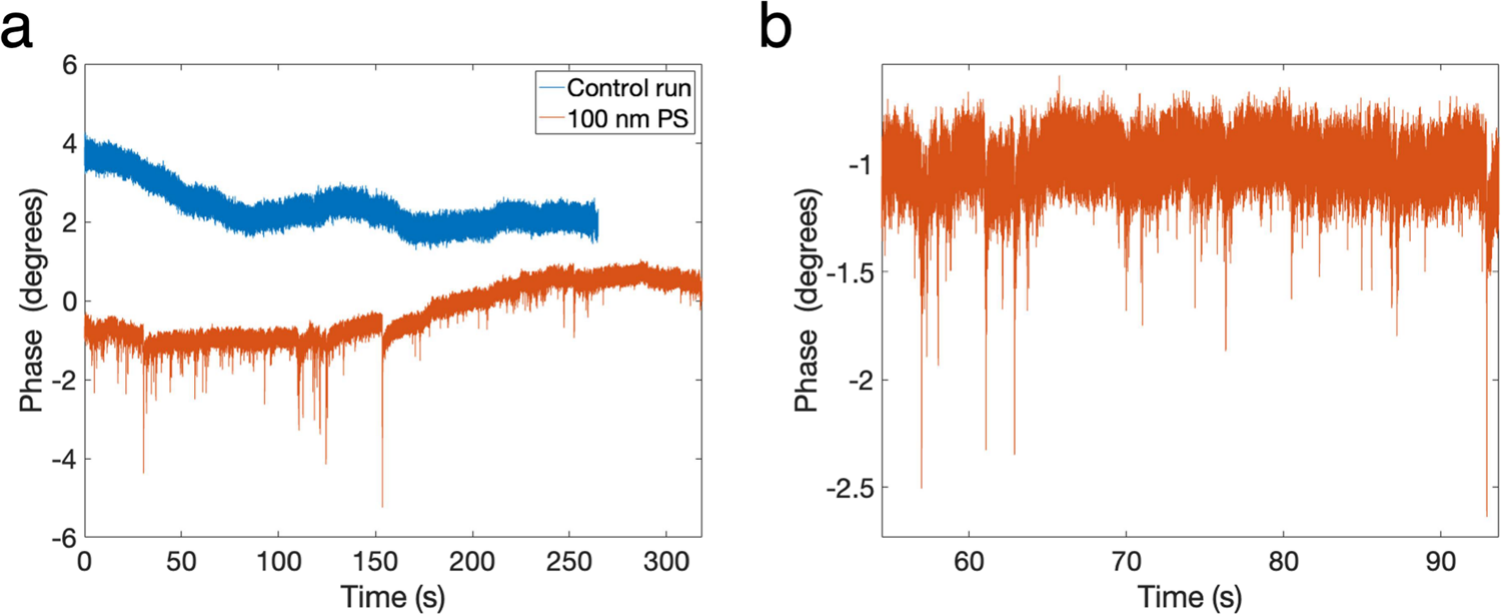
Detecting 100 nm polystyrene particles with a microwave nanopore setup. (a) Control run (blue) and the actual run (orange) with spike-like events. The control run is shown with a 4° phase offset in the *y*-axis so that the data traces do not overlap. (b) Close-up view of some of the events in part (a).

In the experiments, before introducing the nanoparticles into the liquid chamber, control experiments were conducted first, where a PBS solution with no nanoparticles is introduced and 0.5 V was applied to induce electrokinetic motion. The resulting data trace does not contain any sharp features (Figure 3a, blue trace). By contrast, when the 100 nm polystyrene solution was introduced, frequent spike-like transitions were observed typically with several millisecond durations indicative of single-particle translocation events (Figure 3a orange trace, Figure 3b). Ramping up the electrokinetic voltage increased how often these spikes occurred (Figure S5). Since spikes were absent in the control experiments, they were designated to originate from single nanoparticles approaching the sensing region.

**Figure 4:**
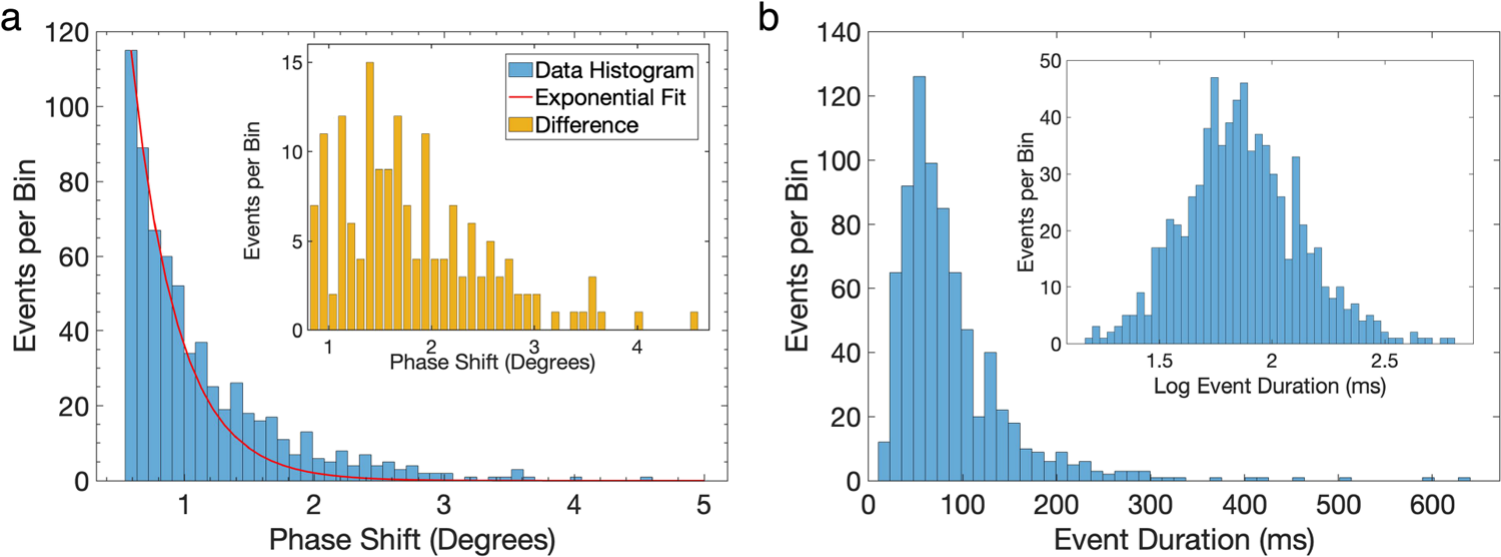
Statistics of 100 nm PS events. (a) Histogram of the frequency shifts is shown as blue bars, whereas the red curve is an exponential fit adjusted with respect to the decay rates of the first few bins. The inset shows the difference between the data histogram and the exponential fit. For clarity, inset event values are rounded up to the nearest integer and reported for events with phase shift > 0.8°. (b) Histogram for the duration of the events. Inset: the same information is shown with a logarithmic *x*-axis.

The histograms for the magnitude of the phase shifts and the event durations were obtained from the data (Figure 4). The histograms were obtained by first filtering the time-series data with a low-pass filter to remove the excess noise (Section S6). The prominence and width values of the spike-like events were determined by the built-in MATLAB functions. A threshold for prominence value was set so that events smaller than the half of the peak-to-peak noise level of control runs were discarded. With this stringent threshold setting, the spike detection algorithm did not register any events in the control runs, as expected. For this reason, all the events detected above the threshold were interpreted to originate from nanoparticles migrating near the active sensing region.

One of the salient features of the phase shift histogram (Figure 4a) is the large number of events at low values and a decaying tail in the distribution. At first, this tail may appear to originate from the noise; however, the stringent criterion for spike detection detailed above and the absence of any events during control experiments indicate otherwise. To further understand this behavior, we fit an exponential curve based on the first few bins of the histogram. After the fitting curve decays to almost zero at large phase shift values, the histogram values remain significantly larger than what is expected from the exponential decay, indicating a secondary population of events. This population—the difference between the original histogram and the exponential fitting—is shown in the inset of Figure 4a. The histogram for the subpopulation has a peak around 1.4° phase shift, which is close to the expected size of the phase shifts for 100 nm PS particles as discussed in the next section.

### Capacitive Changes Induced by Nanoparticles

The measured phase shifts in Figure 4 can be related to the impedance change induced by the nanoparticles, with the real and imaginary parts of the dielectric constant determining the real and imaginary parts of the response, respectively. To obtain the real and imaginary parts, both the amplitude and phase changes of the resonator can be tracked. Within the existing system, only the real part, i.e. the capacitive contribution, of the nanoparticle was accessible (SI Sections S9 and S10), so we focus the discussion on the capacitance change induced by a particle of volume *V*_particle_(28):
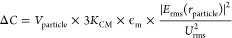
1

where *ϵ*_m_ is the permittivity of the medium (e.g., aqueous solution), *E*_rms_(*r*_particle_) is the root mean squared (rms) electrical field at the particle location, and *U*_rms_ is the rms value of the voltage difference. Finally, *K*_CM_ stands for the Clausius–Mossotti factor which is a function of the permittivity values of the particle (ϵ_p_) and the medium (ϵ_m_):
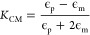
2

Once the expected capacitance is thus obtained by eq 1, it can be first related to the change in the resonance frequency of the sensor, which in turn can be translated into phase shifts by using the phase versus frequency curve of the sensor which is experimentally obtained before the experiments (Supplementary Section S5). This way, the capacitance change induced by nanoparticles can be obtained. We note that the capacitance change depends not only on the properties of the nanoparticle but also on the sensor, such as the distance between the sensing electrodes which determines the local electric field, *E*_rms_(*r*_particle_) at the nanoparticle location.

In the measurements, after subtracting the exponential decay as shown in Figure 4a, the peak value for the phase shift distribution is 1.4°. The slope of the phase versus frequency of the detection circuit is 64.5°/MHz. Thus, a typical nanoparticle event of 1.4° translates into a 21.7 kHz shift in resonance frequency, for a 6.6 GHz resonator. Converting this frequency shift into a fractional capacitance change (), we obtain 6.6 × 10^–6^ fractional change in capacitance induced by a nanoparticle. This value can be compared to the expected capacitance change from eq (1). A 100 nm polystyrene particle (with a relative permittivity of 3.0) passing through an interelectrode distance of 630 nm is expected to induce a 5.4 × 10^–6^ fractional change in capacitance, which is close to the measured value, 6.6 × 10^–6^. Therefore, the size of the observed spikes in this region matches the expected effect of a nanoparticle passing through the nanopore.

**Figure 5:**
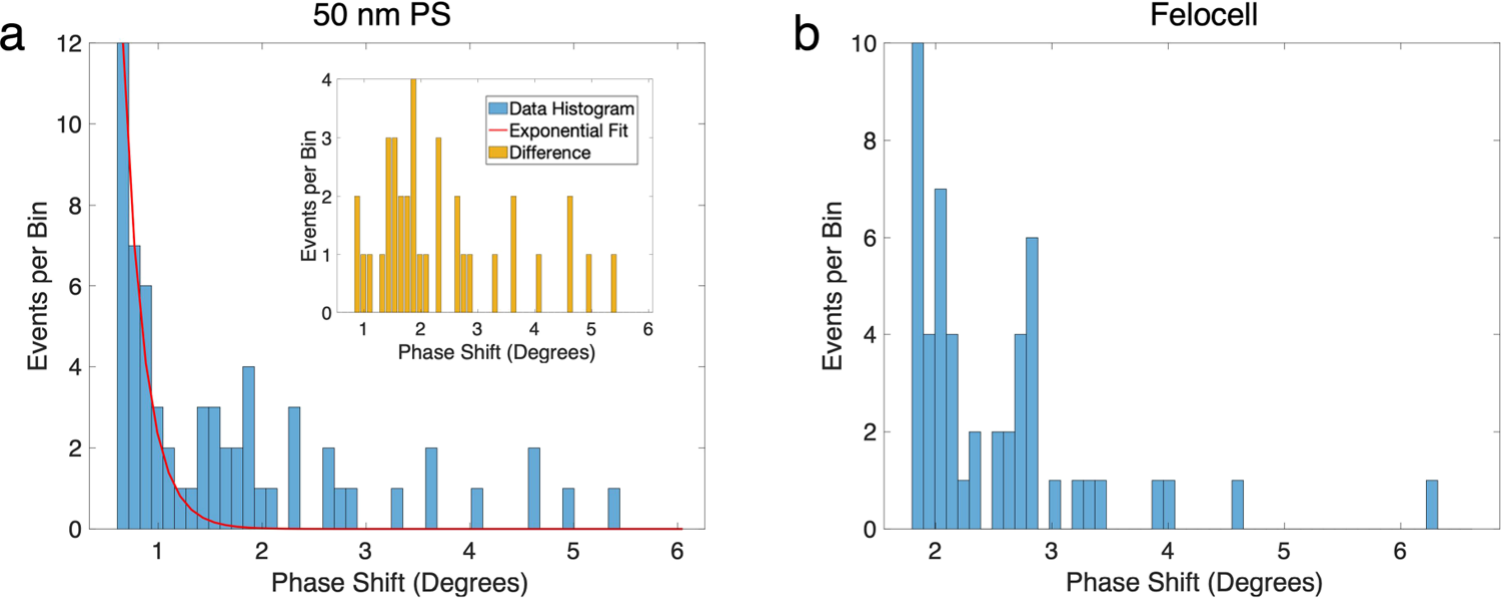
50 nm PS and Felocell vaccine experiments. (a) Histogram of the frequency shifts for 50 nm polystyrene particles. The red curve is an exponential fit adjusted with respect to the decay rates of the first few bins. The inset shows the difference between the data histogram and the exponential fit. For clarity, inset event values are rounded up to the nearest integer and reported for events with phase shift > 0.6°. (b) Histogram for Felocell virions.

To show the broader applicability of the technique, we also tested 50 nm size polystyrene particles and the Felocell vaccine (Zoetis). We used different resonators for each species due to the fragility of the devices (device parameters are listed in SI Section S11). The 50 nm PS particles were diluted by 100-fold, and 25 μL of solution was introduced to the sample reservoir. Felocell vaccine contains virion particles of varying sizes (Feline Rhinotracheitis: 150–200 nm, Calicivirus: 35–39 nm, Panleukopenia: 18–22 nm). The vaccine was in lyophilized form; therefore, it was reconstituted with PBS and filtered with a 220 nm pore-sized filter. After the mixture was pipetted to forestall clustering, 50 μL of viruses in PBS was added to the particle reservoir. Data analysis for both cases were conducted as described before. In both cases, control runs under the same experimental analytes again resulted in data traces with no visible spikes. On the other hand, test runs with the analytes resulted in spike-shaped events and phase shift histograms similar to the 100 nm PS histogram before (Figure 5): a decaying component at lower values and a subpopulation of events at larger frequencies.

The broad nature of the phase shift histogram (Figure 4a) requires further consideration since its large dispersion creates an impediment to a practical sensing scenario. Part of the dispersion may be related to the inherent polydispersity of the sample. A 10 nm standard deviation is reported for 100 nm particles, which translates into a 33% coefficient of variation for the particle volume. While this variation is significant, it cannot, by itself, account for the broadness of the histograms. Here, we consider two additional factors that can contribute to signal broadening: the positional dependence of the sensor response in the nanopore region and particles approaching but not translating through the nanopore.

**Figure 6:**
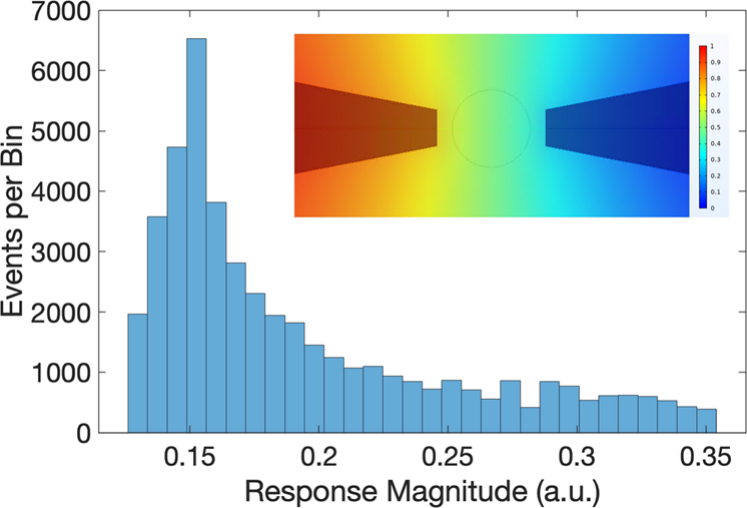
Monte Carlo simulation results for the signal size distribution. The inset shows the electric potential distribution in the sensing region for the simulations, with the outline of the nanopore visible between the two electrodes. The colormap shows voltage levels.

In the device design, the electrode shapes were chosen to be almost triangular to create sharp tips for increasing the electric field intensity (Figure 1e). After nanofabrication, the width of the tip corners appeared to have a finite size of approximately 220 nm, according to scanning electron microscopy (SEM) measurements. Since this width is smaller than the diameter of the nanopore (450 nm), the electric field in the sensing region is not uniform due to the fringing field effects. Since the signal generated by a passing particle is proportional to the square of the electrical field, the response of the sensor depends strongly on where a nanoparticle passes through the nanopore. For instance, the center of the nanopore is aligned with the centers of the electrodes where the electric field is at a maximum: as a result, a nanoparticle passing through the nanopore center will generate a large response. However, the signal magnitude will be smaller for a nanoparticle passing near the edge of the nanopore. We conducted 3D simulations to obtain the overall trend under these conditions (Figure 6, SI Section S7). In this case, the sensing region with the electrodes, membrane, and nanopore was modeled inside water to obtain the electric potential and field (Figure 6, inset). Using the extracted electric field magnitudes, a Monte Carlo simulation was conducted to calculate the response of the nanoparticles, assuming a uniform distribution through the nanopore (excluding edges with a margin of 50 nm so that the nanoparticles can fit). The response of each particle was calculated as the integral of the square of the electrical field for the volume of each 100 nm particle (at the vertical position where their response is maximal). The result qualitatively captures that a large portion of the signals occurs at lower values, which decay out with a tail at high values. Thus, the electrical inhomogeneity and positional dependency of the sensor partially explain the decaying trend in the experimental histogram.

A second potential factor for the broad distribution of the events is that a nanoparticle can still induce a signal even if it does not translocate through the nanopore: it is sufficient that they migrate to a region with a large enough electric field strength. The field near the nanopore region is large enough to result in a nanoparticle signal above the noise floor but not large enough to induce full response that would have occurred if the particle passed through the center of the nanopore. Such incidents may further contribute to the number of events, giving rise to signals at the lower values of the histogram. By contrast, a resistive-pulse sensing technique requires the particle to be present inside the nanopore, so that a blockage current can be generated. This requirement is an advantage for obtaining uniform signals in resistive-pulse sensing. However, the proximal sensing capability of capacitive sensors can offer an advantage in studying the dynamics of nanoparticles and other analytes as they approach the nanopore.

We note that, even with a uniform electric field, the capability for material classification requires the attainment of very high signal-to-noise ratio in microwave sensing.^19^ This requirement is due to the fact that the Clausius–Mossotti factor depends weakly on the particle permittivity, when the permittivity of the medium (e.g., water) is large. In the case of water with dielectric permittivity approaching 80, the Clausius–Mossotti factors for most materials are close to −1/2. The similarity in Clausius–Mossotti factors for different materials constitutes a problem for material classification. One exception in this case is the classification between nonbiological versus biological particles; since biological particles contain water in their structure, and so they can attain values significantly different than −1/2. For the challenging task of differentiating between nonbiological particles, in addition to attaining larger signal-to-noises ratio, changing the type of the medium could be a potential solution. By changing water with a low-permittivity liquid, the contrast difference between different particles can increase, albeit an overall reduction in signal magnitude is expected to occur since the microwave signal change in eq 1 has an overall multiplication factor for the permittivity of the medium.

In this work, we operated the system only at a single frequency, which provides only parameters at that frequency. To obtain multifrequency data, either a broadband measurement or multiplexed narrowband detection^30,31^ could be employed in future work. In the analysis of nanoparticle data (SI Section S5), only the capacitance change of the bare particle was considered; the ionic shell surrounding the particle was ignored for the sake of simplicity. Due to the fragility of the nanomembrane devices, we had to use different devices in different runs. Due to the nonuniformity of the electrical field (e.g., Figure 6, inset), device-to-device variations especially in the active region, and the uncertainty in the sensitivity analysis (SI Section S8), no attempt was made to compare the different sample runs with each other at this stage. While microwave interferometer topology increases the sensitivity, it is not entirely clear whether this topology also introduced extra noise on the measurements.

The stage of development for microwave nanoparticle sensors can be compared to the early days of nanomechanical mass spectrometry,^32,33^ as well as impedance cytometry,^34^ where position dependency of the analytes conspired to broaden the signal values. Later on, the use of multiple modes in nanomechanics^35,36^ and multielectrode approaches in impedance cytometry^37−39^ was invented to factor out positional effects. Similarly, the results obtained here motivate the development of new device designs and measurement techniques to eliminate the positional dependency of nanoparticle signals for capacitive sensing. Beyond single nanoparticle sensing reported here, microwave capacitive sensing can be applied to dynamically characterize ion distributions inside nanoscale channels to provide a novel characterization technique for nanofluidics.^40−42^ Orthogonal measurement modalities can also be seamlessly integrated with microwave sensors, such as by incorporating UV-absorbing nanomaterials for liquid characterization,^43^ integrating flexible membranes for fluid flow measurements,^26,44^ and coupling to mechanical resonators for deflection-based mass determination.^45^

## Conclusions

In conclusion, we demonstrated proof-of-concept experiments for capacitive sensing of nanoparticles as they migrate close to a nanoscale sensing region and translocate through a nanopore. The measurements are conducted by tracking the phase response of a microwave resonator. As a particle modulates the capacitance of the microwave resonator, the resonator phase is modulated as well, which is detected by custom measurement circuitry. The measured phase shift can be related first to the capacitance change induced by the particle and then to its electrical size. Capacitive sensing is critical to provide both material-dependent information in electronic sensing and understanding the transient dynamics of analytes as they approach the nanopore region. With the proof-of-principle demonstration of on-chip microwave sensing, the stage is ripe for dielectric sizing and characterization of nanoparticles capacitively in liquids.

## Supporting Information Available

Schematic of the experimental setup, fabrication scheme, VNA response of a microwave resonator, microwave circuit, time traces for particle and control experiments, phase response of the resonator, sensitivity analysis, and devices used in the experiments (PDF)
