# Supplementary material for: Dielectric Detection of Single Nanoparticles Using a Microwave Resonator Integrated with a Nanopore
Source: ACS Omega. 2024 Feb 8;9(7):7827–34. doi: 10.1021/acsomega.3c07506 (PMC10882703; doi:10.1021/acsomega.3c07506)
Supplement: Supplementary file 1 — ao3c07506_si_001.pdf [file ao3c07506_si_001.pdf]

Supplementary Information for

Dielectric Detection of Single Nanoparticles Using a Microwave Resonator Integrated with a Nanopore

Arda Secme[1,2,†,‡], Berk Kucukoglu[1,2,†,‡], Hadi S. Pisheh[1,2,‡], Yagmur Ceren Alatas[1,2], Uzay Tefek[1,2] Hatice Dilara Uslu[1,2] , Batuhan E. Kaynak[1,2], Hashim Alhmoud[1,2], M. Selim Hanay[1,2,]*

[1]Department of Mechanical Engineering, and

[2] UNAM – Institute of Materials Science and Nanotechnology,

Bilkent University, Ankara, 06800, Turkey

S1. Device Fabrication

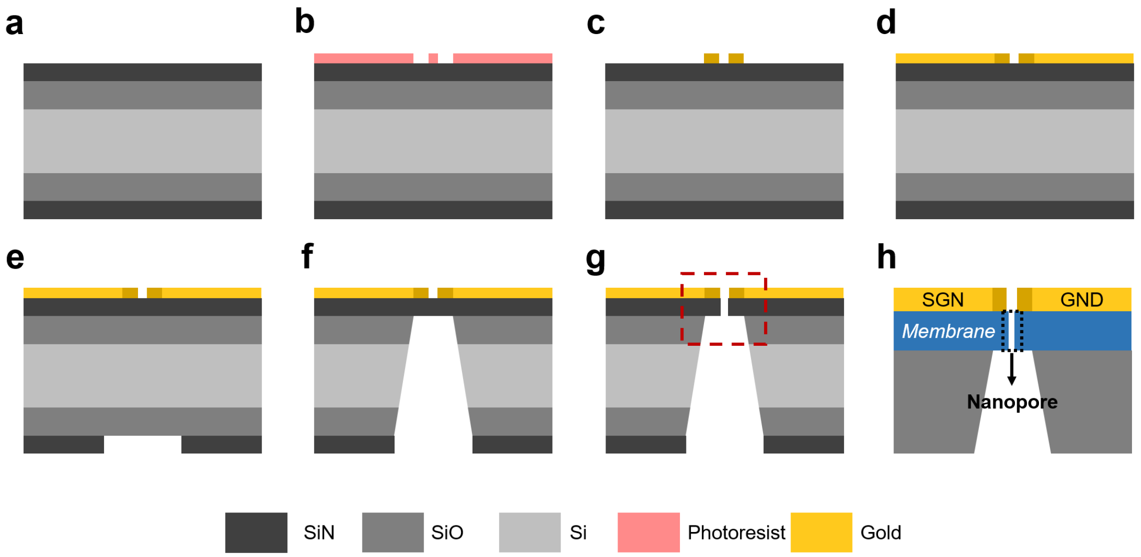

Figure S1: Fabrication Scheme.

Gold electrodes were patterned in two steps: First, electron beam lithography (EBL) was performed to pattern the gold electrodes in the sensing region separated by a gap of hundreds of nanometers. Thermal metallization was used to deposit 10 nm thick chromium stiction layer and 100 nm thick gold electrodes. In the second lithography step, larger features of electrodes were aligned with the electrodes in the first step and this time photolithography was performed and the chip was again metallized with gold using thermal evaporation.

The formation of nanopore starts with releasing the membrane beneath the gold electrodes. First, nitride at the bottom of the chip was etched anisotropically using inductively coupled plasma etching. Then, KOH wet etch was performed to etch through the chip until the top nitride layer which forms the suspended membrane. In the last step, focused ion beam was employed to open a hole on the membrane which is the nanopore around the gold electrodes.

S2. Experimental Setup for I-V Curve

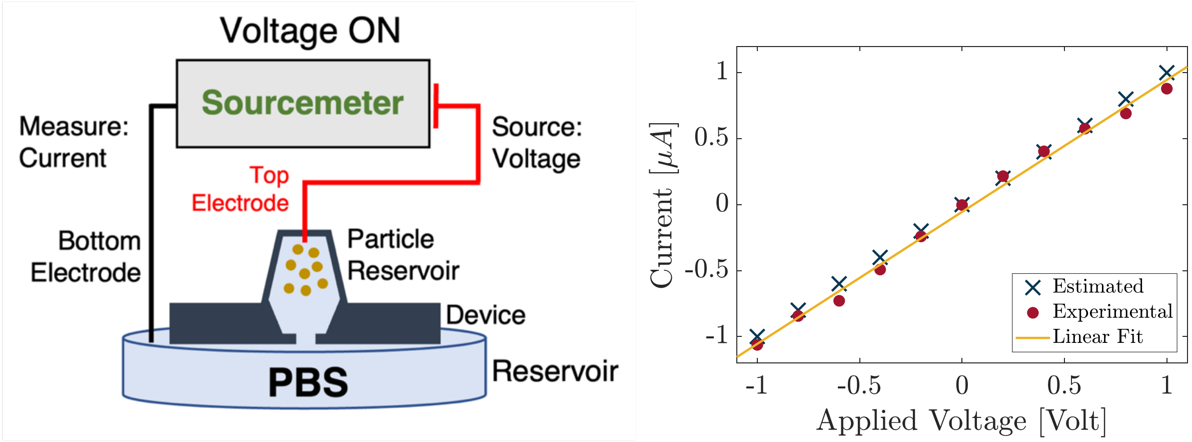

Figure S2: Left Panel: Schematic of the experimental setup for I-V measurements.
Right Panel: A typical I-V curve.

For applying the electrokinetic motion, we employed a sourcemeter as a voltage source and a current reader simultaneously. The potential difference between the two electrodes drives particles to pass through the nanopore, then the I-V curve of the device is plotted as shown below. This experiment was performed before any nanoparticle experiment.

S3. Vector Network Analyzer Response & Measurement Circuitry

Initial characterization of the microwave resonator was conducted using a vector network analyzer which is shown below. We note that, the network analyzer measurements are conducted to find the location of the resonance before actual nanoparticle sensing experiments. During the nanoparticle sensing measurements, a custom build circuitry based on microwave interferometry[1, 2] is used (Section S5), the output of which possesses a different phase variation trend than what is observed in the VNA.

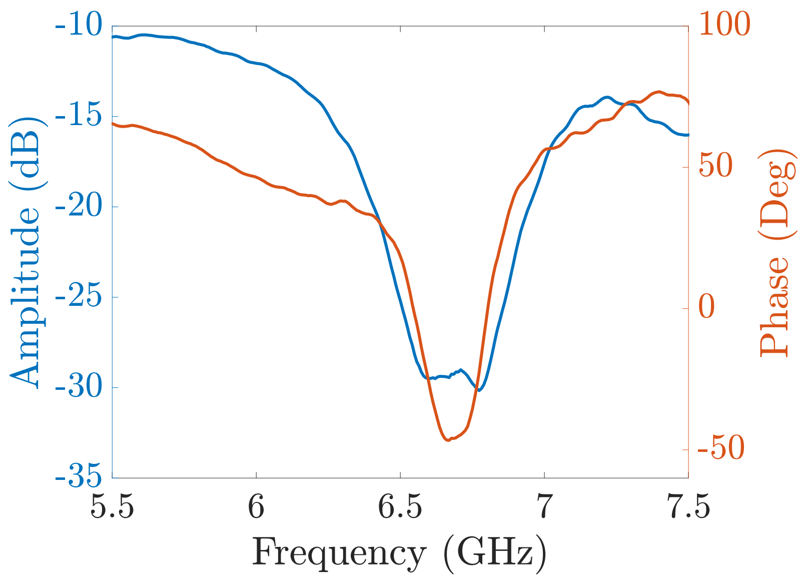

Figure S3: VNA response of a microwave resonator.

Since the designed CPW resonator is a single port device, we could only check the S11 (reflection) response. However, for both VNA measurements and experiments, we placed an RF Circulator before the port. With the circulator, we can drive the resonator from the first port of the circulator and then read its reflection response at the third port. The custom circuit used in the experiments is shown below which produces a heterodyne detection for the microwave resonator.

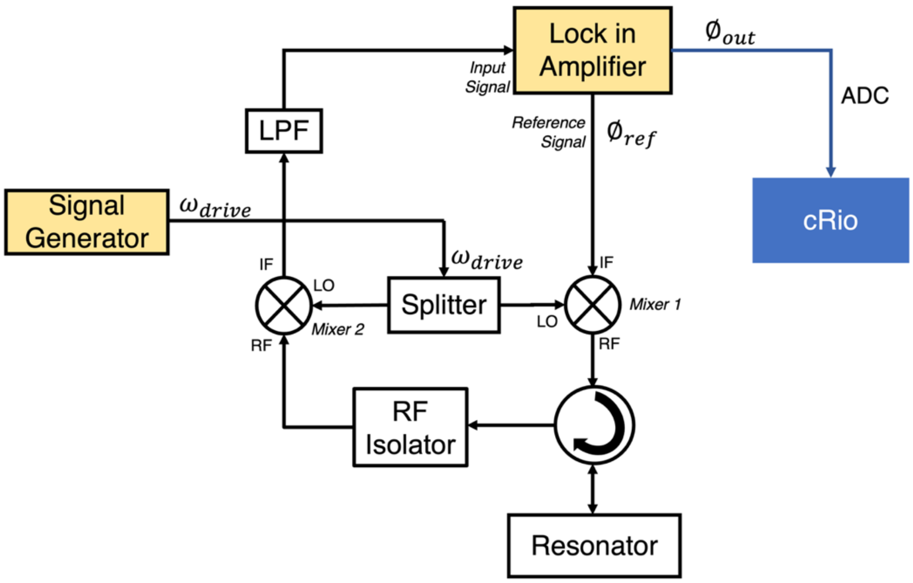

Figure S4: Microwave circuit used in nanoparticle sensing experiments. LPF: low-pass filter, ADC: Analog to digital converter

The microwave signal from signal generator (ω_{drive} ~ 6.6 GHz) was first split into two branches. One branch was up converted at Mixer 1 with the reference signal from the lock in amplifier ($\Delta\omega$ ~ 3 MHz). This signal then propagated to the circulator to drive and read the response of the resonator. An RF Isolator was placed to reduce the interference due to reflections. After the isolator, the response was down converted at Mixer 2 using the second branch of the original microwave signal. The mixing products were filtered with a low pass filter (4.5 MHz) and fed into the locking amplifier. The output of this detection circuit block, $\emptyset_{out}$, is the phase difference between the instantaneous phase and the reference phase at the resonance frequency of the device. There is no feedback between the output and input signal so that the resonator dwells around its resonance frequency with a slow drift. For high speed measurements, the phase output from the lock in amplifier was collected using cRio (NI) analog input module.

S4. Increasing Event Rate by Increasing Translocation Voltage

To show control over the electrokinetic motion, we changed the applied DC voltage. The blue traces are control runs and there are no precipitous jumps induced by particle translocation. However, on the red traces, as we increased the electrokinetic voltage, we can observe that spikes occur more frequently as the voltage is increased.

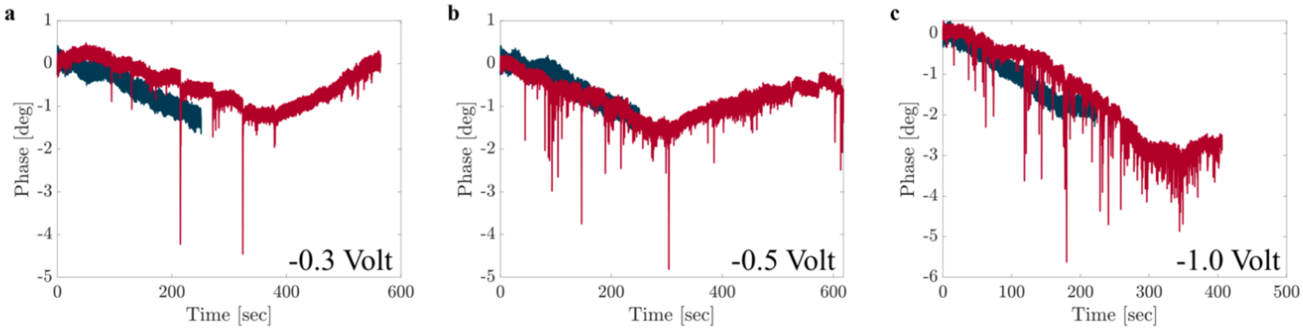

Figure S5: Time traces for particle (red) and control (blue) experiments as a function of different translocation voltages.

S5. Capacitance Change Calculation

The parameters for the macroscopic section of the CPW resonator are illustrated below (the drawing is not to the scale):

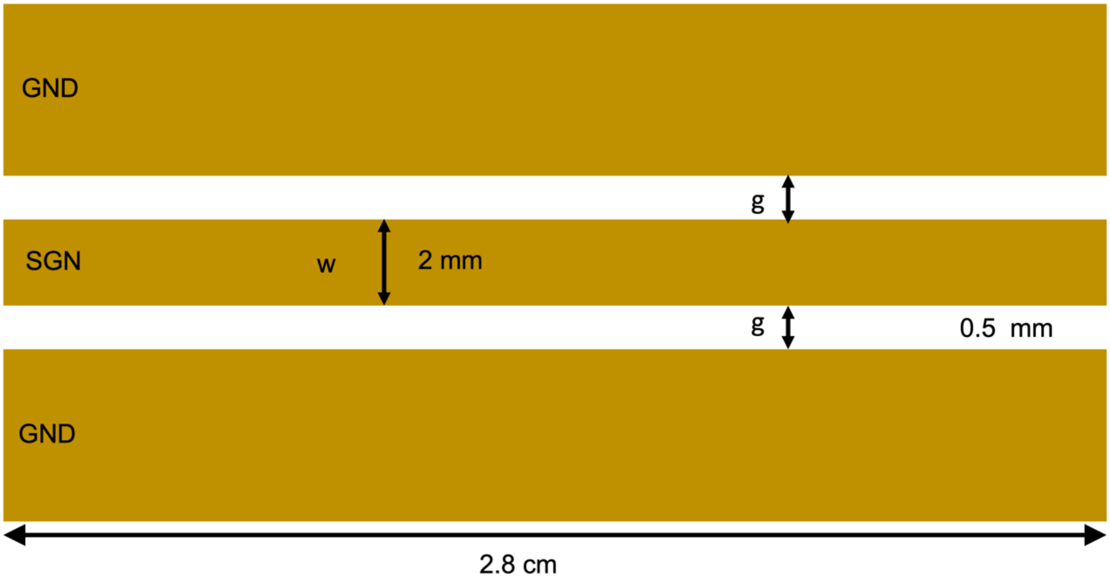

Figure S6: Distances in the macroscopic section of the CPW resonator.

Here, the width of the transmission line is 2 mm and there is a gap between signal and ground which is 0.5 mm. The parallel section of the coplanar waveguide until the bowtie shape is 2.8 cm.

By using a standard numeric calculator for CPW transmission lines, we obtain Cs=0.67 pF / cm, thus for a 2.8 cm length, C_{CPW}=1.9 pF is obtained. Since mode shape is not uniform across the resonator structure, we need to account for the variation by integrating the mode shape squared along the CPW to obtain the effective capacitance. This integral provides a ¼ multiplier to account for the mode shape. Thus, effective capacitance becomes C_0=0.48 pF.

Next we calculate the capacitance change induced by a 100 nm PS particle by using equation 1 in the main text.

$$\Delta C = V_{particle} \times 3\, K_{CM} \times \epsilon_m \times \frac{\left|E_{rms}(r_{particle})\right|^2}{U_{rms}^2}$$

Here we take distance between the electrodes as **g** in the sensing region (this is 630 nm for the device used for taking 100 nm PS data). When a particle passes through this region through a nanopore, the geometry of the sensing electrode is similar to a parallel plate capacitor. Thus, we can approximate:

$$E_{rms}\left(r_{particle}\right) \approx \frac{U_{max}}{g}$$

Where U_{max} is the maximum voltage which is at the sensing region (assuming that the resonator is well designed so that the sensing region is located at an antinode of electric field). On the other hand U_{rms} through the sensor is $U_{rms} = \frac{U_{max}}{\sqrt{2}}$. Therefore, the last factor is:

$$\frac{\left|E_{rms}(r_{particle})\right|^2}{U_{rms}^2} = \frac{2}{g^2}$$

Then we will have

$$\Delta C = V_{particle} \times 3\, K_{CM} \times \epsilon_m \times \frac{2}{g^2}$$

$$\Delta C = \frac{4}{3}\pi(5\ 10^{-8}\ m)^3 \times 3\left(-0.47 \times 78 \times 8.85\frac{10^{-12}F}{m}\right)\frac{2}{(630\ 10^{-9}m)^2} = 2.6\ 10^{-18}F$$

Thus, the expected capacitance change is approximately 2.6 aF.

For the device used in Figure 2 (6.61 GHz) the gap size is 630 nm and for a 100 nm particle we have:

$$\frac{\Delta C}{C_0} \sim \frac{2.6\ aF}{0.48\ pF} \sim 5.4\ 10^{-6}$$

The phase vs frequency response of the detection circuit is shown below. The maximum slope extracted in the transition region is 64.5 deg/ MHz at the resonance frequency, 6.62 GHz. Thus a 5.4 ppm level capacitance change will result in 17.8 kHz frequency shift ($\Delta f = \frac{f_0}{2}\frac{\Delta C}{C_0}$). We multiply this with 64.5 deg/MHz and we get 1.2 degrees phase shift for 100 nm PSNP which is close to

what we observe in experiments (1.4 degrees). We also note that there is a certain degree of uncertainty in the determination of the slope in the sharp transition region due to the low number of data points obtained during the original frequency sweep; indeed, a smaller slope of 18.5 deg/MHz can be extracted by using the other two data points in the transition, which is 3.5 times smaller than 64.5 deg/ MHz.

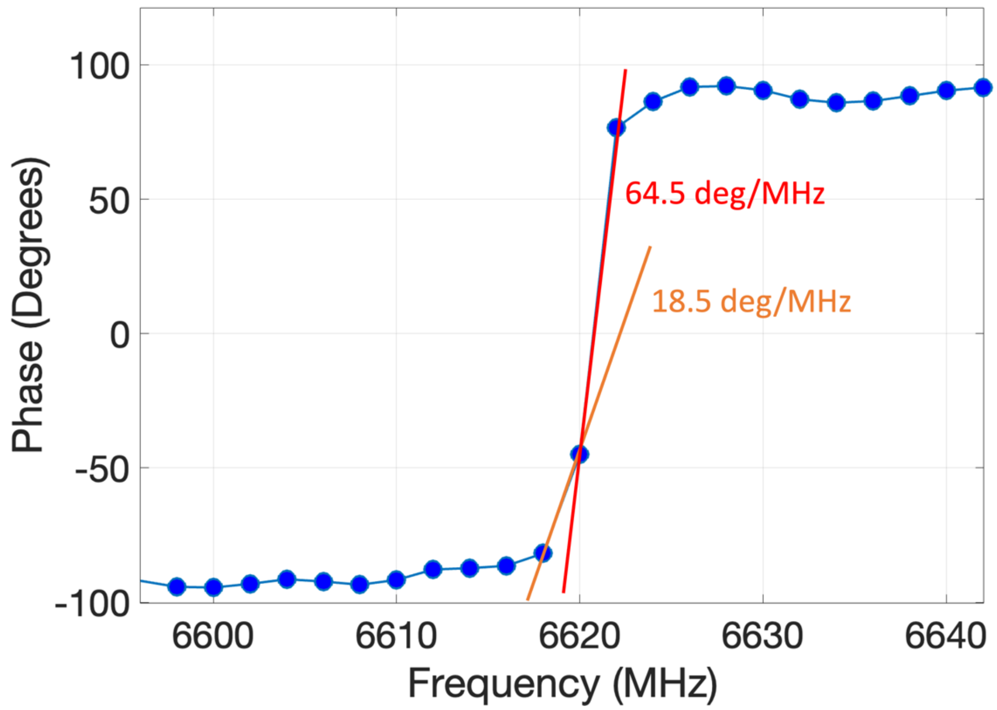

Figure S7: Phase response of the resonator in the measurement circuitry.

S6. Filtering for Data Analysis

Data analysis relies on peak-finding algorithms. These algorithms can be sensitive to high-frequency noise such as those caused by power lines. Therefore, a lowpass filter is used to smoothen the data. The filter is a standard Chebyshev Type II filter with a passband and stopband of 25Hz and 35Hz, respectively. It has a 0.5dB passband ripple, 60dB stopband attenuation, and is designed using MATLAB Filter Designer application. The designed filters are applied to data using built-in MATLAB functions which do not introduce phase shifts.

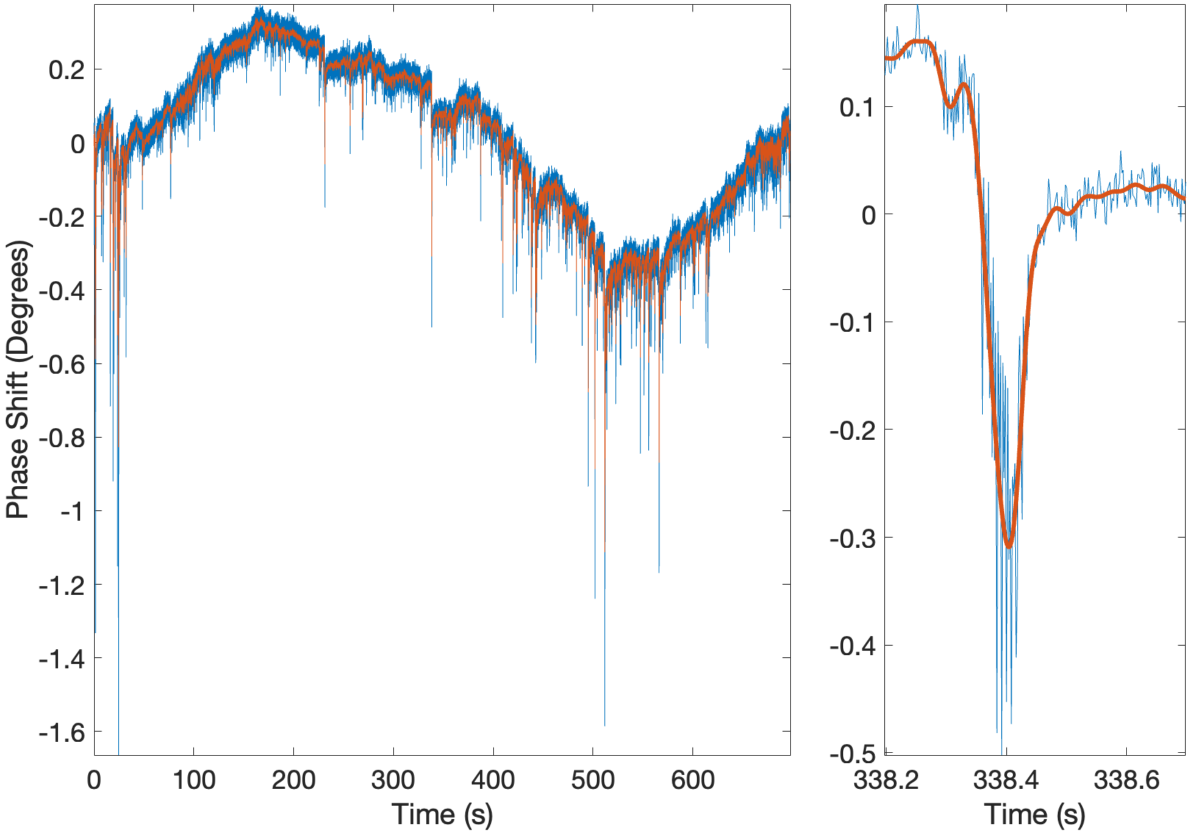

Figure S8: Close-up view of an experimental data set and particle passage, raw data (blue) and low pass filtered data (orange).

On the left, there is an example of raw and filtered datasets. Lowpass filtering of the dataset significantly reduces the high-frequency noise content, resulting in cleaner datasets for peak-finding algorithms to work on. It can be seen clearly with an emphasis on a particular event.

S7. Numerical Simulations for Position-Dependent Response

The simulations were conducted in COMSOL Multiphysics 5.6 using Electrostatics module to obtain the potential energy distribution and electric field near the nanoscale sensing region of the coplanar waveguide resonator which exhibits a quasi-TEM mode. The simulation space was limited to a 8 µm by 2 µm by 2 µm volume centered at the mid-point between the electrodes. This way part of the Silicon Nitride membrane and the whole nanopore was included in the

simulation volume. The maximum mesh size was set to 20 nm resulting in 69 million mesh elements. After the study was simulated, the electric field distribution between the electrodes were exported to MATLAB for further analysis (with a uniform grid spacing of 5 nm). Here, the particle responses were evaluated with a Monte Carlo approach for their in-plane position (x,y); but their vertical position was fixed at z=0 i.e. when vertically at the mid-point of the nanoscale electrodes – since this is the location where they induce maximum response during they trajectory. For the in-plane Monte Carlo simulations, all the positions on the nanopore radius were assumed to be equally likely as long as the nanoparticle fits inside the nanopore. This means that the 100 nm diameter nanoparticles in the simulations had an at least 50 nm gap is imposed between the nanopore boundary. In experiments, particles approaching to nanopore from the periphery may still pass through it and contribute further to the events at low signal values. For each particle, the response is calculated as the square of the electrical field was numerically summed for a sphere of diameter 100 nm centered at the particle location. The signal responses were represented as a histogram with 25 bins.

S8. Sensitivity Analysis

It is of interest to check how much the capacitance signal changes, as a function of change in the permittivity value of the particle (i.e. conduct sensitivity analysis). To do so, we first plotted the K_{cm} value (which is proportional to the capacitance signal) with respect to the permittivity of the particle as shown on the right. Different curves correspond to different choices of medium.

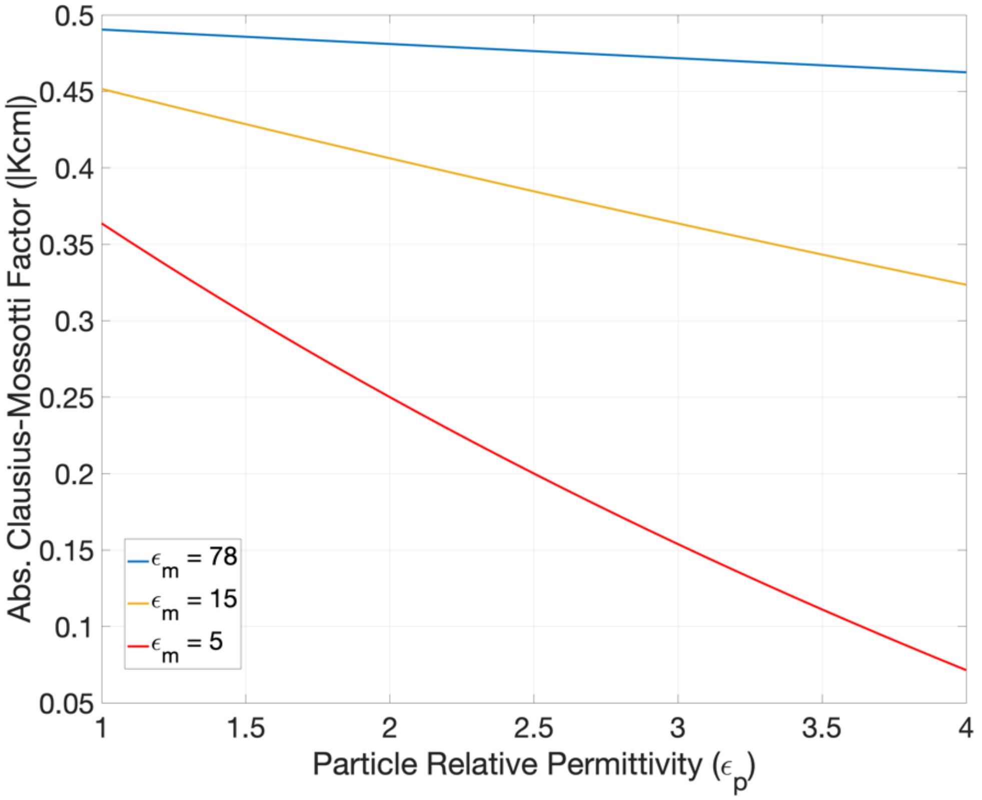

Figure S9: Change of Clausius-Mossotti factor as a function of the particle and medium permittivity (different colors show the situation for different values of medium permittivity).

In the current work, we had opted to work with water (blue curve, with relative permittivity of 78) due to its prevalence in biology and environment. As evident from the graph, the slope of the curve is very small for water which indicates a limitation for the technique. For different choices of the permittivity (e.g. for sensing applications where there is a freedom for the operation liquid), the sensitivity (i.e. the slope) increases drastically, especially when the permittivity of the medium is close to the permittivity of the particle, as is the case with the red curve, where the medium permittivity is 5.

For the three different values of medium permittivity, we have listed the % of change for a 10% change in dielectric permittivity of the particle.

Permittivity of the Medium	% Change in Capacitance for a 10% change in the permittivity of the particle (for $\epsilon_p = 2.2$)
5	% 8.3
15	% 2.1
78	% 0.4

Table S1: Sensitivity of the capacitance with respect to the permittivity of the medium.

So when working with water, a 10% change in particle permittivity will translate only to 0.4% change in capacitance signal; whereas, if the medium was changed as to have a permittivity of 5, then the change in capacitance signal would be comparable (%8.3 for Kcm vs. %10 for permittivity).

S9. Capacitive and Resistive Response Considerations for Nanoparticles

The microwave resonator used in the experiments can be modeled as a series RLC circuit. When the nanoparticle passes through the sensing region, it introduces a combination of a capacitance change of ΔC and a resistance change of ΔR, due to the real and imaginary parts of its dielectric permittivity, respectively.

$$\Delta C = 3\, V_{particle} \times Re\{K_{CM}\} \times \epsilon_m' \times \frac{\left|E_{rms}(r_{particle})\right|^2}{U_{rms}^2}$$

$$\Delta R = 3\, V_{particle} \times Im\{ K_{CM}\} \times \epsilon_m'' \times \frac{\left|E_{rms}(r_{particle})\right|^2}{U_{rms}^2}$$

In the measurement circuits, the response of the circuit can be expressed as a transfer function in frequency domain. For a single port design like our CPW, the transfer function corresponds to the ratio of reflected wave voltage V^- to the incident wave voltage V^+. This ratio is also defined as S_{11} parameter. This parameter is a function of line impedance of the measurement circuitry, and input impedance of the device Z_{in}.

$$H(\omega) = \frac{V^-}{V^+} = S_{11} = \frac{Z_{in} - Z_0}{Z_{in} + Z_0}$$

For and RLC circuit the transfer function can be written as,

$$H(\omega) = \frac{(R + \Delta R - Z_0) + j\left(\omega L - \frac{1}{\omega(C + \Delta C)}\right)}{(R + \Delta R + \ Z_0) + j(\omega L - \frac{1}{\omega(C + \Delta C)})}$$

Since the sensor is driven at its resonance frequency (ω_0), the impedance of the sensor has to be purely real. Therefore, at resonance.

$$\omega_0 L - \frac{1}{w_0 C} = 0$$

Also, if use Taylor Expansion to the capacitance term to the first degree;

$$\frac{1}{\omega_0(C + \Delta C)} = \frac{1}{\omega_0 C} - \frac{1}{\omega_0 C}\left(\frac{\Delta C}{C}\right)$$

Then, the transfer function at resonance frequency can be written as:

$$H(\omega) = \frac{(R + \Delta R - Z_0) + j\frac{1}{\omega_0 C}\left(\frac{\Delta C}{C}\right)}{(R + \Delta R + Z_0) + j\frac{1}{\omega_0 C}\left(\frac{\Delta C}{C}\right)}$$

We note that for a series RLC circuit, $Q = \frac{1}{RC\omega_0}$ thus we can simplify the above expression:

$$H(\omega) = \frac{(R + \Delta R - Z_0) + jQR\left(\frac{\Delta C}{C}\right)}{(R + \Delta R + Z_0) + jQR\left(\frac{\Delta C}{C}\right)}$$

Next, we divide the numerator and denominator by R , and we define:

$$z_0 \equiv \frac{Z_0}{R}$$

$$\delta r \equiv \frac{\Delta R}{R}$$

$$\delta c \equiv \frac{\Delta C}{C}$$

With these definitions, the transfer function can be written as:

$$H(\omega) = \frac{(1 + \delta \mathrm{r} - z_0) + jQ\,\delta c}{(1 + \delta \mathrm{r} + z_0) + jQ\,\delta c}$$

If the expression is multiplied with its complex conjugate and it's real and imaginary parts are separated.

$$H(\omega) = \frac{[(1 + \delta \mathrm{r})^2 - z_0^2 + Q^2\,\delta c^2]\; + j2Qz_0\,\delta c}{(1 + \delta \mathrm{r} + z_0)^2 + Q^2\,\delta c^2}$$

We note that in the experiments Q values are typically 20-50 whereas the δc (normalized capacitance change) values are smaller than 10^{-4} . Thus we can deduce $Q\,\delta c \ll 1$ and we can ignore the quadratic term $Q^2\,\delta c^2$ at the nominator and denominator since there are terms with lower order in the perturbation such as a term in $O(\delta r)$. This way, the transfer function becomes:

$$H(\omega) = \frac{[(1 + \delta \mathrm{r})^2 - z_0^2]\; + j2Qz_0\,\delta c}{(1 + z_0 + \delta \mathrm{r})^2}$$

With this transfer function, we can easily separate the real (X-quadrature) and imaginary (Y-quadrature) components of the transfer function. It is more instructive to start with the imaginary component:

$$Y \equiv Im\{H(\omega)\} = \frac{Q\,z_0}{(1 + z_0 + \delta \mathrm{r})^2}\,\delta c$$

We can now see that, regardless of the matching condition of the circuit (i.e. the value of $z_0 = \frac{Z_0}{R}$) the imaginary component of the signal is linearly proportional to the capacitance change induced by the particle. This comes about because the inductive and capacitive components of the RLC

circuit cancel each other at resonance, and any additional capacitance perturbation is not hampered by a background value.

For the above expression, we can also observe that, the δr term in the denominator is much smaller than the remaining $(1 + z_0)$ term, and it can be ignored. With this simplification, the final expression for the Y-quadrature becomes:

$$Y \equiv \frac{Q\, z_0}{(1 + z_0)^2}\, \delta c$$

Now returning back to X quadrature, we can write the real part:

$$X \equiv Re\{H(\omega)\} = \frac{(1 + \delta\mathrm{r})^2 - z_0^2}{(1 + z_0 + \delta\mathrm{r})^2}$$

We then simplify this expression by ignoring terms $O(\delta r^2)$ and carrying out the algebra:

$$X = \frac{1 - z_0^2 + 2\, z_0\, \delta r}{(1 + z_0)^2}$$

Here, we realize that unlike the case of Y, the term containing δr (*i.e.,* $2\, z_0\, \delta r$) does not show up in the response proportionally, but rather is added to another term ($1 - z_0^2$) which may be much larger (with the notable exception of $z_0 = 1$ *i.e.* when the resistance of the RLC resonator happens to match the line impedance). **Thus, for the general case, the resistive contribution of the nanoparticle is buried with the larger response of the dissipative component of the resonator. For this reason, unless a special precaution is taken, the resistive contribution is not accessible with the existing architecture.**

In the experiments reported here, we work with the phase of the resonator (ϕ). The phase can be expressed as the arctangent of the ratio of the imaginary and real parts of the response:

$$\phi = \arctan\left(\frac{Y}{X}\right) \approx \frac{Y}{X}$$

where the last step is written since Y is a small quantity ($O(\delta c)$). Thus we have:

$$\phi = \frac{Q\, z_0}{1 - z_0^2}\ \delta c$$

Reverting this expression back to unnormalized parameters:

$$\phi = \frac{Q\ R\ Z_0}{R^2 - Z_0^2}\left(\frac{\Delta C}{C}\right)$$

As expected, the phase of the resonator is linearly proportional to the added capacitance of the nanoparticle, which is measured in the experiments.

$$\phi = \frac{\frac{2Z_0}{\omega_{res} C}}{(R^2 - Z_0^2)}\left(\frac{\Delta C}{C}\right)$$

Thus, tracking phase changes will essentially track the capacitance change, i.e. the real part of the dielectric constant.

S10. Necessary Conditions for Measuring the Resistive Change of Nanoparticles

Following the discussion on section S9, it may be instructive to see how the resistive effect of the nanoparticles can be measured. First, we need to ensure that the resistive effect is not buried by the background response by satisfying $R = Z_0$. (We remind that R in this case is the value of the resistance for the RLC series model of the microwave resonator). In this case:

$$X = \frac{\delta r}{2} = \frac{\Delta R}{2R}$$

$$Y \equiv \frac{Q\ Z_0}{4R}\left(\frac{\Delta C}{C}\right)$$

In this case (where R=Z_0), a detection circuit which can **separately detect X and Y components can extract both the resistive (ΔR) and capacitive (ΔC) effects of a nanoparticle.**

If the phase of the resonator is calculated in this specific case:

$$\phi = \arctan\left(\frac{Y}{X}\right) = \arctan\left[\frac{Q}{2}\frac{\left(\frac{\Delta C}{C}\right)}{\left(\frac{\Delta R}{Z_0}\right)}\right]$$

Note that the variable inside is no longer small, so we cannot directly perform a Taylor expansion. To further operate on the expression, we copy the resistive and capacitive contribution terms:

$$\Delta C = 3\, V_{particle} \times Re\{K_{CM}\} \times \epsilon'_m \times \frac{\left|E_{rms}(r_{particle})\right|^2}{U_{rms}^2}$$

$$\Delta R = 3\, V_{particle} \times Im\{\, K_{CM}\} \times \epsilon_m{}'' \times \frac{\left|E_{rms}(r_{particle})\right|^2}{U_{rms}^2}$$

We see that most of the parameters are the same. In terms of K_{cm} values, when water is used as the medium at microwave frequencies, both the real and imaginary K_{cm} values are similar (-0.5). Thus, the ratio $\Delta C/\Delta R$ yields $\frac{\epsilon'_m}{\epsilon''_m}$ for the (*aqueous*) medium. We can rewrite the inside as

$$\phi = \arctan\left(\frac{Y}{X}\right) = \arctan\left[\frac{Q\, Z_0}{2C}\,\frac{\epsilon'_m}{\epsilon''_m}\right]$$

Note that the effects of resistance and capacitance nulls each other, and phase does not change in this case, even if there is a particle passage for this specific case. Thus, to resolve the resistive and capacitive changes separately, in addition to operating at a specific impedance (R=Z_0), the detection circuitry should be able to provide X and Y quadratures independently.

S11. Devices used in the Experiments

Analyte	Resonance Frequency of the Sensor	Distance between electrodes	Nanopore Hole Size
**100 nm PS**	6.61 GHZ	630 nm	450 nm
**Felocell**	5.19 GHZ	860 nm	460 nm
**50 nm PS**	7.19 GHZ	1500 nm	700 nm

References

1. Ferrier, G. A.; Romanuik, S. F.; Thomson, D. J.; Bridges, G. E.; Freeman, M. R. A Microwave Interferometric System for Simultaneous Actuation and Detection of Single Biological Cells. Lab on a Chip 2009, 9, (23), 3406-3412.
2. Nikolic-Jaric, M.; Romanuik, S.; Ferrier, G.; Bridges, G.; Butler, M.; Sunley, K.; Thomson, D.; Freeman, M. Microwave Frequency Sensor for Detection of Biological Cells in Microfluidic Channels. Biomicrofluidics 2009, 3, (3), 034103.
